# Large animal models of ischemic mitral regurgitation—systematic review and meta-analysis

**DOI:** 10.3389/fmedt.2025.1687873

**Published:** 2026-01-15

**Authors:** Christian D. Andreasen, Tanita D. Jeppesen, J. Michael Hasenkam, Johannes H. Jedrzejczyk

**Affiliations:** 1 Department of Cardiothoracic and Vascular Surgery, Aarhus University Hospital, Aarhus, Denmark; 2 Department of Clinical Medicine, Aarhus University, Aarhus, Denmark

**Keywords:** cardiac disease, ischemic mitral regurgitation (IMR), large animal models, mitral valve (MV), translational medicine

## Abstract

**Background:**

Ischaemic mitral regurgitation (IMR) is a significant complication of myocardial infarction associated with increased morbidity and mortality. Large animal models are essential for testing novel mitral valve therapies, yet no consensus exists on the optimal infarction strategy to induce IMR.

**Methods:**

A systematic review and meta-analysis were conducted to evaluate infarction strategies used to induce IMR in large animal models. Studies were identified through database searches and screened according to predefined inclusion criteria. Subgroups were stratified by infarction strategy. Proportions of IMR development and severity were analysed using a random-effects model, and reporting quality was assessed across studies.

**Results:**

Forty-four studies met the inclusion criteria, comprising 869 animals across 52 subgroups. Ethanol injection in select obtuse marginal arteries (EtOH-OMx strategy) yielded the highest rate of IMR development (87%, 95% CI: 79%–96%) with the lowest associated mortality. Ligation of obtuse marginal arteries under cardiopulmonary bypass (CPB-OM2, OM3 and CPB-OMx) demonstrated high mortality and inconsistent IMR severity. Reporting quality was variable, with frequent omissions regarding sex, randomisation, and adverse event documentation.

**Conclusions:**

This review identifies the EtOH-OMx strategy as a promising method for inducing IMR in large animal models, demonstrating favourable performance within the limitations of available data.

## Graphical Abstract

**Figure:**
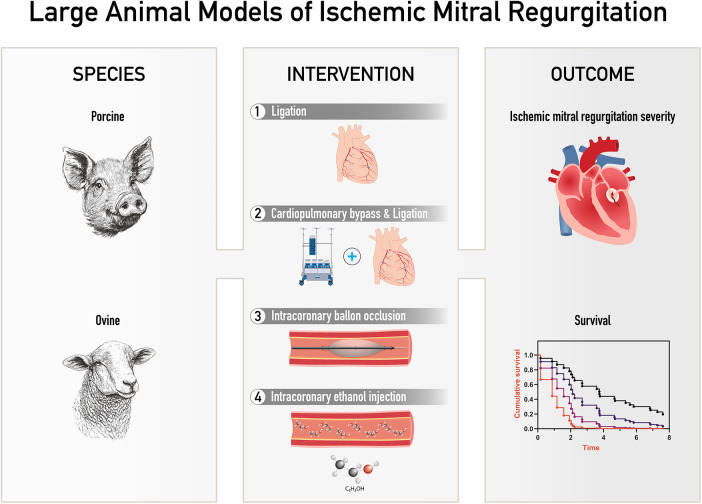

## Introduction

Ischemic mitral regurgitation (IMR) is a secondary form of mitral regurgitation caused by left ventricular dysfunction, remodelling, and displacement of the papillary muscles. Although the mitral leaflets and chordae tendineae are structurally intact, they become functionally compromised due to altered ventricular geometry. This results in an imbalance between tethering forces (exerted through the chordae tendineae) and closing forces, leading to incomplete leaflet coaptation and regurgitation (1–3).

The incidence of IMR following acute myocardial ischemia is reported to range from 17% to 58% (4–6). Even mild IMR is associated with an increased risk of heart failure and reduced survival compared to patients without IMR (4, 5, 7–9). Current clinical guidelines from both the ACC/AHA and ESC/EACTS recommend medical therapy as first-line treatment for patients with severe secondary mitral regurgitation and heart failure, with cardiac resynchronisation therapy advised in select cases (10, 11). Surgical intervention is recommended only in specific patient subsets, according to the ACC/AHA guidelines (10). Surgical treatment of IMR remains challenging, with high perioperative mortality and a substantial risk of recurrent regurgitation at follow-up (12–14). These challenges are compounded by patient heterogeneity and the lack of a standardised surgical approach or device. While novel transcatheter mitral valve replacement systems have shown promising early results (15), further validation is needed.

Preclinical trials are crucial for evaluating the safety and efficacy of emerging mitral valve repair and replacement techniques. These studies are typically conducted in large-animal models of mitral regurgitation, which share anatomical and physiological features with the human heart (16). The first large animal model of IMR, developed by Llaneras et al. (17)], has been widely adopted. Subsequent models have been introduced to improve our understanding of IMR pathophysiology and to assess novel therapeutic approaches. Despite these developments, there is currently no established gold standard for inducing IMR in large animals. The objective of this systematic review is to identify the most effective method for IMR induction and to determine which model achieves the highest rate of successful IMR development.

## Methods

This study followed the PRISMA (18) guidelines for systematic reviews and meta-analyses. The review protocol was not prospectively registered in PROSPERO, OSF, or other registries.

### Search string, article identification and screening

The search string consisted of three terms: “mitral regurgitation”, “animal models”, and “relevant animals” (see Supplementary Table S1). PubMed and Embase were used to identify relevant studies. The search had no time restriction but was restricted to English publications. The final search was conducted in November 2024. Articles were imported into EndNote X9 and Covidence, where they were automatically screened for duplicates. PubMed and Embase were selected because they provide broad coverage of cardiovascular, surgical, and preclinical research in large animal models. However, omitting additional databases such as Scopus and Web of Science may have led to the exclusion of relevant studies. Title, abstract, and full-text screening were conducted in Covidence. Criteria for exclusion were: follow-up <4 weeks, natural history of animals, *ex vivo*/*in vitro*, systematic reviews, unavailability of full-text, conference abstracts, no echocardiographic quantification of ischemic mitral regurgitation, and non-relative measurements of ischemic mitral regurgitation. Criteria for inclusion were: ischemic mitral regurgitation in native mitral valves, follow-up >4 weeks, and echocardiographic quantification of ischemic mitral regurgitation. Studies could contribute one or more subgroups with disparate infarction strategies if the subset(s) conformed to the inclusion and exclusion criteria. Studies demonstrating the induction of ischemic mitral regurgitation and its subsequent amelioration were eligible at the time point when ischemic mitral regurgitation was present, and the other criteria were met. A *post hoc* exclusion of subgroups was conducted to exclude articles that used non-ischemic methods to induce mitral regurgitation in animals.

### Quality of reporting assessment

The studies were evaluated using a self-developed questionnaire based on SYRCLE's risk-of-bias tool for animal studies (19–21). We developed a bespoke reporting-quality questionnaire that combined selected items adapted from the SYRCLE risk-of-bias tool with additional criteria specific to large-animal surgical models. The SYRCLE-derived items addressed reporting of baseline characteristics, allocation methods, blinding, and outcome assessment, while the additional items focused on transparency of perioperative procedures, adverse-event reporting, and echocardiographic methodology. A complete list of questionnaire items, including their origin (SYRCLE-derived vs. additional), is provided in Supplementary Table S2. As our tool was designed to evaluate completeness of reporting rather than to assign domain-level risk-of-bias judgements, no overall risk-of-bias rating was generated; instead, item-level frequencies were summarised across all included studies.

The results were reported collectively using dichotomous responses (“yes” or “no”), expressed as a percentage of the total number of included studies (0%-100%). A “no” response primarily indicated that the item was not reported in the manuscript or Supplementary Materials and should not be interpreted as definitive evidence that the procedure was not performed. Reporting quality was categorised as good (>75%), average (50%–75%), or poor (<50%). Two questions were further subdivided and analysed in separate tables based on the initial “yes” or “no” responses. All included studies, including their Supplementary Materials and appendices, were thoroughly reviewed to extract all available information.

### Data extraction

Studies could contribute to one or more subgroups, depending on the techniques used. Studies that did not clearly report mortality or the presence or absence of ischemic mitral regurgitation were excluded from data extraction and from the quantitative synthesis. We did not contact the original study authors to obtain missing data, as the heterogeneity and age of the studies made consistent retrospective clarification unlikely. Studies that did not comment on death or lack transparency regarding the outcome of the animals did not undergo data extraction. If there had been no deaths and the authors had acknowledged this, the study would have been eligible for data extraction. All studies and subgroups were identified, and the following data were extracted: animal type, number and sex of animals, infarction strategy, procedural outcomes (death, no ischemic mitral regurgitation, or development of mitral regurgitation), and the method used to quantify ischemic mitral regurgitation. The accepted quantification methods for ischemic mitral regurgitation are the regurgitation fraction (RF%) and the percentage of regurgitation jet of the left atrial area (RJ/LAA%), which offer the advantage of being relative measurements. Thus, measurements are independent of the animal's size. All outcome data were collected at the eight-week time point or the closest time point.

### Data analysis

The proportion of animals developing IMR was assessed through meta-analysis. Subgroups were eligible if they included ≥2 animals, and each infarction method required at least two qualifying subgroups to be included in the analysis. These thresholds were selected to ensure that effect estimates were based on minimally interpretable sample sizes and to avoid generating unstable estimates for infarction strategies represented by only single or small subgroups.

For each subgroup, the proportion (p) of animals developing IMR was calculated as (number with IMR/total number of animals), along with the corresponding standard error (SE). The infarction strategy then pooled the subgroups, and weighted effect estimates were calculated using a random-effects model in EpiBasic v4.4 (22), expressed as *p* ± SE. This analysis reflects the reported proportion of IMR development as interpreted by the original studies. If a study explicitly stated that IMR was achieved in a subset of animals, that proportion was used. Results were visualised as bar charts showing proportion estimates with 95% confidence intervals. *P*-values were calculated using EpiBasic v4.4, with the infarction technique described by Llaneras et al. (17) serving as the reference group. No formal correction for multiple comparisons was applied, and the resulting *p*-values should therefore be interpreted with caution; effect sizes and their confidence intervals represent the primary basis for interpretation.

The severity of IMR in each subgroup was classified using an ordinal scale: none, mild, moderate, and severe, based on echocardiographic criteria from the American Society of Echocardiography (23) with no regard to the original author's own interpretation of IMR severity. Because no validated severity criteria exist for large-animal IMR, the human guideline criteria were used as conceptual guides rather than strict numerical thresholds. Relative measures (RJ/LAA% and RF%) were therefore interpreted in relation to the qualitative structure of the Zoghbi framework (23), without applying human absolute cut-offs to animals. When studies used non-standard thresholds or qualitative terms, we mapped their definitions to our four-category scale (none, mild, moderate, severe) based on their relative positions to guideline-based criteria and the grading scheme used by the original authors. This approach ensured consistency without imposing arbitrary human-specific limits.

An additional category, “dead”, was included to account for mortality before assessment. All animals within each subgroup were stratified according to these categories, ensuring that every subject was represented. IMR severity was visualised as a cumulative bar chart showing the proportion of animals in each severity category for each infarction strategy. Outcomes classified as dead, none, or mild were considered negative, whereas moderate and severe were considered positive. Severity distributions were also tabulated by study within each infarction strategy and qualitatively assessed for inter-study heterogeneity. The random-effects model was fitted using the DerSimonian-Laird method for estimating between-subgroup variance (τ^2^). Proportions were analysed on the raw scale without logit or Freeman-Tukey transformation. Standard errors were calculated from binomial variance, and 95% confidence intervals were constructed using normal approximation. These choices preserved interpretability and reflected common practice in preclinical meta-analyses with moderate group sizes. All analyses were performed in EpiBasic v4.4.

Formal heterogeneity statistics, such as *I*^2^ and *χ*^2^, were not calculated because most infarction strategies included very few subgroups, resulting in insufficient degrees of freedom for meaningful or interpretable estimates. Under these conditions, these statistics are known to be unstable and can produce misleading values. Heterogeneity was therefore assessed qualitatively by comparing severity distributions graphically.

## Results

### Literature search and screening

**Figure 1:**
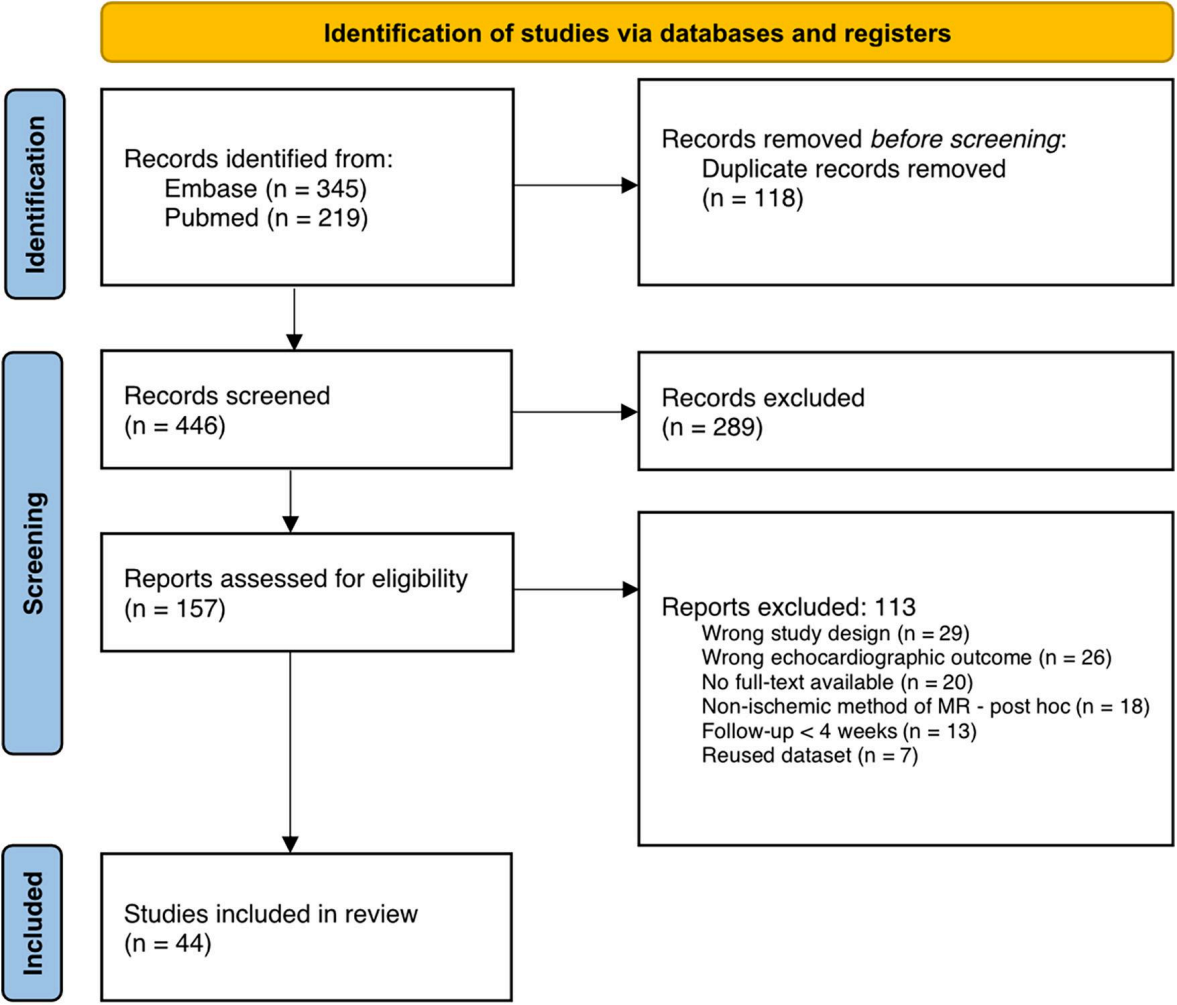
Article identification and screening flowchart adapted from PRISMA guidelines.

The database search identified 564 records, of which 446 remained after duplicates were removed. Following title and abstract screening, 157 articles were assessed in full text. Of these, 113 were excluded because they did not meet the inclusion criteria. A total of 44 studies were included in the final review (17, 24–66). The study selection process is outlined in Figure 1.

### Study characteristics

The 44 included studies comprised 52 animal subgroups undergoing infarction procedures, involving a total of 869 animals. Only two species were used: pigs (*n* = 236, 27%) and sheep (*n* = 633, 73%). Sex was reported in 41% of studies, with male animals comprising 74% of the reported cases. Follow-up duration ranged from 4 to 16 weeks, with a mean of 8.04 weeks per subgroup. The mean follow-up time per infarction strategy ranged from 6.5 to 9.1 weeks.

### Infarction strategies

**Figure 2:**
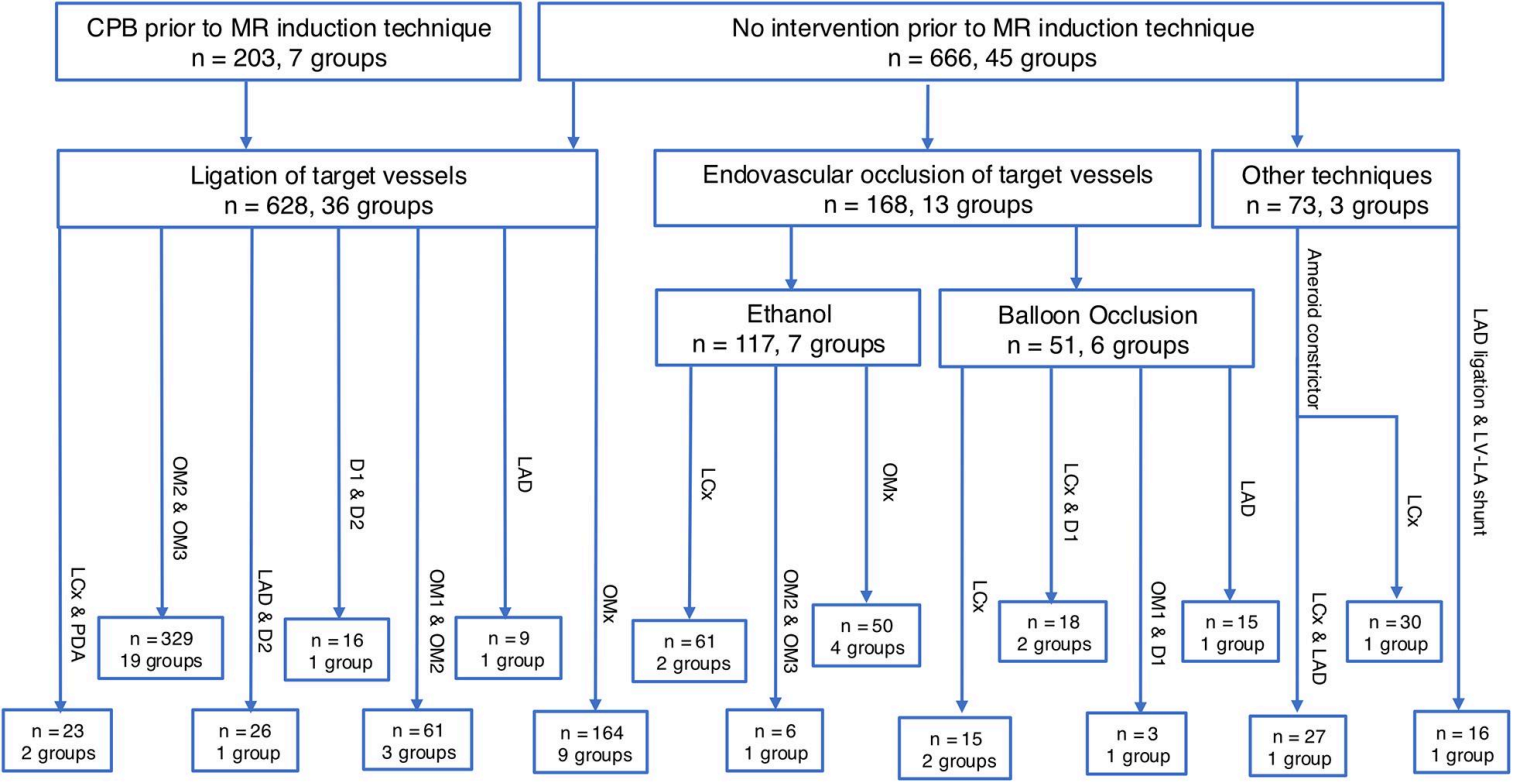
Overview of infarction strategies of ischemic mitral regurgitation, divided into vessel targets. Seventeen infarction strategies were identified. CPB, cardiopulmonary bypass; D1, D2, diagonal arteries 1 and 2; LAD, left anterior descending artery; LCx, left circumflex artery; LV-LA, left ventriculoatrial; MR, mitral regurgitation; OM1, OM2, OM3, obtuse marginal arteries 1, 2, and 3; OMx, select obtuse marginal arteries; PDA, posterior descending artery.

Figure 2 depicts an overview of the infarction methods. In seven subgroups (*n* = 203/869, 23.3%), cardiopulmonary bypass was established prior to the induction of ischemic mitral regurgitation through a left thoracotomy. All subgroups received implantation of positional markers, and snares were placed around target arteries and exteriorised through the skin to allow for follow-up investigations. The animals were weaned from cardiopulmonary bypass and closed, and after 8 ± 2 days, the snares were tightened under general anaesthesia.

Twenty-nine subgroups (*n* = 425/869, 49%) underwent ligation of target coronary arteries via left or right thoracotomy. Snares were placed around the target vessels and tightened to achieve complete occlusion. The OMx ligation strategy, performed with or without cardiopulmonary bypass, is based on the anatomical identification of the middle and lateral cardiac veins, between which the target arteries are located. Ligation and subsequent occlusion of these arteries induced infarction (37, 40, 46, 50–53, 62, 63).

Seven subgroups (*n* = 117/869, 13%) underwent percutaneous ethanol (EtOH) injection into target coronary arteries under fluoroscopic guidance. A balloon catheter was advanced to the selected vessel and inflated at the intended site, followed by the infusion of 1–5 mL of ethanol. This procedure was repeated until all target vessels had been treated. Percutaneous EtOH injection is also used in combination with the OMx strategy. This approach involves identifying the posteromedial papillary muscle and its arterial supply, either through simultaneous ventriculography and angiography (55, 56) or through contrast-enhanced echocardiography during selective perfusion of the target arteries with radiographic contrast (33, 34).

Six subgroups (*n* = 51/869, 6%) utilised a percutaneous balloon occlusion approach, in which a balloon catheter was advanced to the target vessel, its position confirmed by fluoroscopy, and the artery occluded by balloon inflation for 90–120 min. ST-segment elevations were observed in all subgroups, confirming successful infarction.

Three studies employed alternative infarction strategies that did not fit the predefined categories and are therefore not further discussed (24–26).

### Assessment of reporting quality

**Figure 3:**
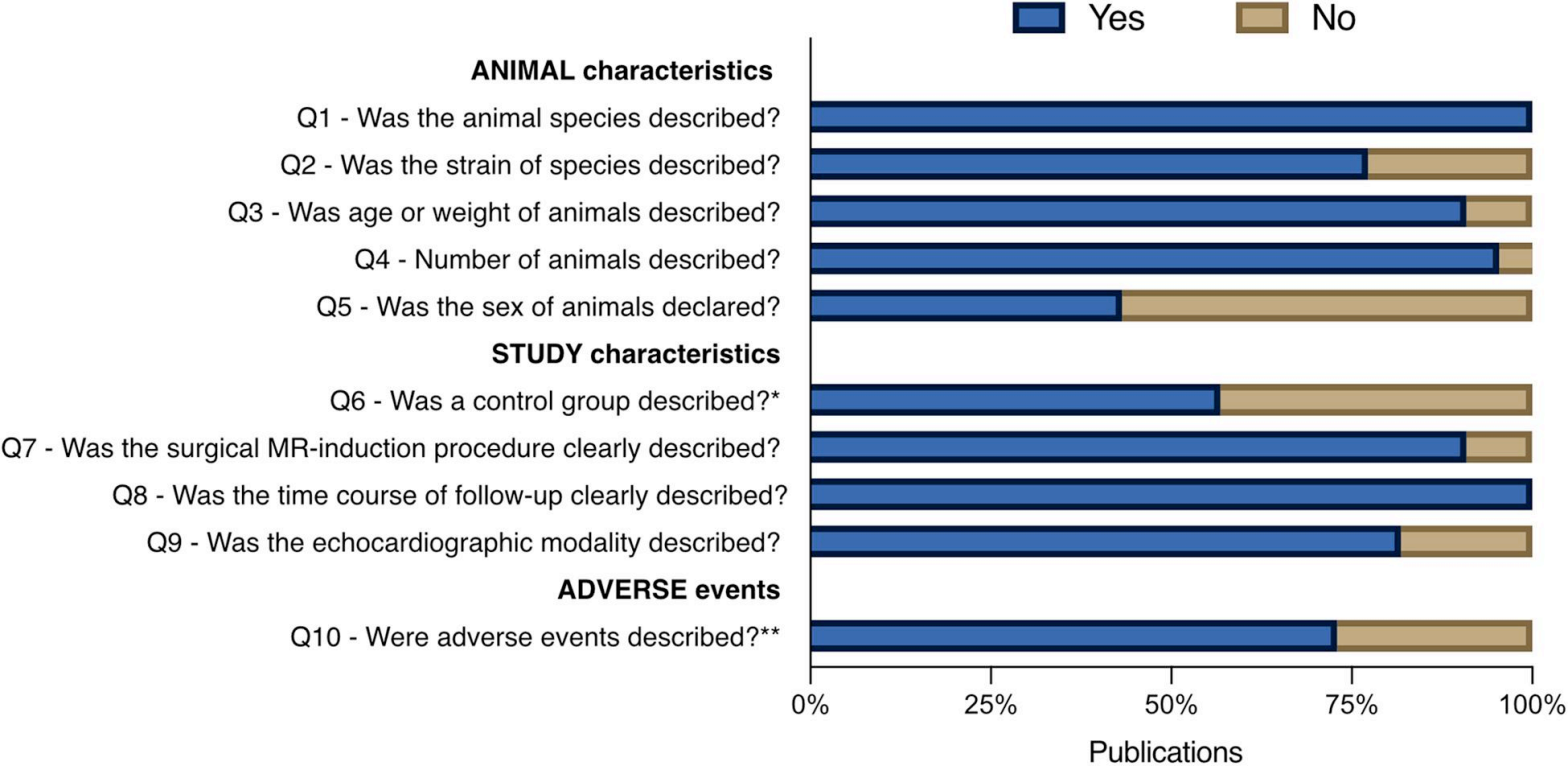
Quality of reporting assessment. Questions Q1–Q5 address reporting of animal characteristics, Q6–Q9 address study design elements, and Q10 concerns the reporting of adverse events. *Details related to Q6 are presented in Table 1; **details related to Q10 are presented in Table 2.

Figure 3 summarises the assessment of reporting quality. Animal characteristics (Q1–Q5) were generally well reported, except sex, which was specified in only 43% of studies (19/44). Study characteristics (Q7–Q9) were also well documented. In contrast, Q6, concerning the use and description of control groups, was only moderately reported, with 57% of studies (25/44) providing relevant information. Given the heterogeneity of study designs, Q6 is further detailed in Table 1. Among the 25 studies that reported a control group, randomisation was described in only 44% (11/25), and blinding of the final echocardiographic assessment was reported in 36% (9/25). Of the 19 studies that did not include a control group, 26% (5/19) aimed to develop a novel IMR model, while 74% (14/19) were feasibility or valve geometry studies. Adverse events were moderately reported overall, described in 73% of studies (32/44). Within this subset, the timing and allocation of events were well documented in 78% (25/32) and 81% (26/32), respectively. In the remaining 27% (12/44), adverse events were either not reported or the reporting was deemed insufficiently transparent, raising concerns about potential omissions in animal inclusion or exclusion (37).

**Table 1:** Continuation of Q6. “Yes” and “No” answers in Q6 are categorised into categories to explore the control groups.

Question	Yes	No
*Q6) Was a control group described?	25 (56.8%)	19 (43.2%)
In case yes:		
Was the number of animals per group described?	23 (92.0%)	2 (8.0%)
Was randomization of animals into groups present and described?	11 (44.0%)	14 (56.0%)
Were there comparable baseline values for groups?	19 (76.0%)	6 (24.0%)
Was the final echocardiographic assessment done blinded?	9 (36.0%)	16 (64.0%)
In case no:		
No, the study is a MR-model study?	5 (26.3%)	
No, the study is a feasibility/valvular geometric study?	14 (73.7%)	

**Table 2:** Continuation of Q10. “Yes” and “No” answers are categorised to explore adverse events.

Question	Yes	No
*Q10) Were adverse events or no adverse events clearly stated?	27 (61.4%)	17 (38.6%)
In case yes:		
Were the allocation of adverse events clearly described in a group(s)?	23 (85.1%)	4 (14.8%)
Was the time point of adverse events clearly described?	25 (92.6%)	2 (7.4%)
In case no:		
No, the author fails to “comment on adverse events”	14 (82.4%)	
No, suspicion arise to author not including diseased animals in article	3 (17.6%)	

### Meta-analysis

A total of 20 studies were excluded from the meta-analysis for methodological reasons (see Figure 2). Twelve studies were excluded due to a lack of transparency regarding mortality or failure to report outcome data (26, 30–32, 37, 39, 43, 45, 52–54, 57). One study was excluded because it had fewer than two animals per subgroup, rendering it ineligible for analysis (64). The remaining seven studies were excluded due to an insufficient number of eligible subgroups per infarction strategy, which prevented statistical aggregation of data for those methods (24, 25, 27, 38, 44, 59, 66).

#### Stratification of ischemic mitral regurgitation development based on infarction strategy

**Figure 4:**
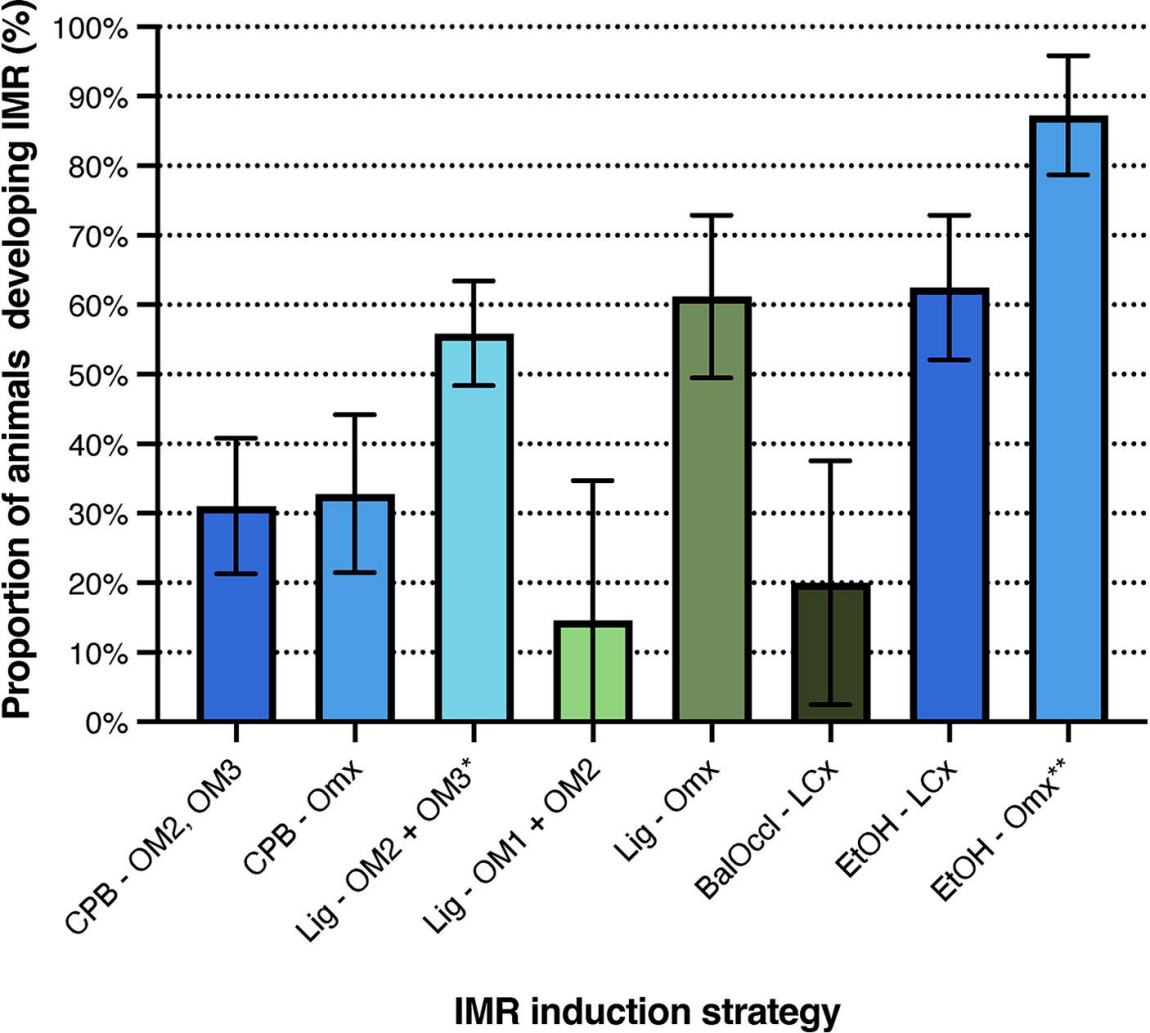
Meta-analysis of ischemic mitral regurgitation development stratified by infarction strategy, with the IMR proportion as determined by the original authors. Results are visualised as mean proportions ±95% confidence interval. BalOccl, balloon occlusion; CPB, cardiopulmonary bypass; EtOH, ethanol; LCx, left circumflex artery; Lig, ligation; OM1, OM2, OM3, obtuse marginal arteries 1, 2, or 3; OMx, select obtuse marginal arteries. *Reference infarction strategy. **p < 0.05.

Data were extracted from 24 studies comprising 26 subgroups and stratified into seven distinct infarction strategies for IMR induction (see Figure 4). Notably, all non-endovascular strategies were performed exclusively in sheep. In contrast, endovascular approaches were conducted solely in pigs, except for the EtOH-OMx strategy, in which 1 of 4 subgroups (25%) involved sheep.

Results are reported as the original authors' estimate of IMR development. The infarction strategy originally described by Llaneras et al. (Lig-OM2, OM3) resulted in IMR in 56% of animals [95% CI (48%–63%)] (16, 28, 29, 41, 42, 48, 58) and served as the reference group. Comparable outcomes were observed for the Lig-OMx (40, 46, 50, 51) and EtOH-LCx (47, 65) strategies, with no statistically significant difference (*p* > 0.05). In contrast, the CPB-OM2, OM3 (49, 60, 61), CPB-OMx (62, 63), Lig-OM1, OM2 (16, 48), and Balloon Occlusion-LCx (35, 36) strategies demonstrated markedly lower pooled proportions of IMR development, with confidence intervals that did not overlap the reference method. The EtOH-OMx strategy (33, 34, 55, 56) achieved the highest success rate, inducing IMR in 87% of animals [95% CI (79%-96%), *p* < 0.001]. When the single sheep subgroup (33) was excluded, the IMR incidence remained high at 85% [95% CI (76%–95%), *n* = 48; 3 subgroups], with statistical significance retained across all comparisons (*p* < 0.001). For detailed numerical values and comparisons, see Supplementary Table S3.

#### Stratification of ischemic mitral regurgitation severity based on infarction strategy

**Figure 5:**
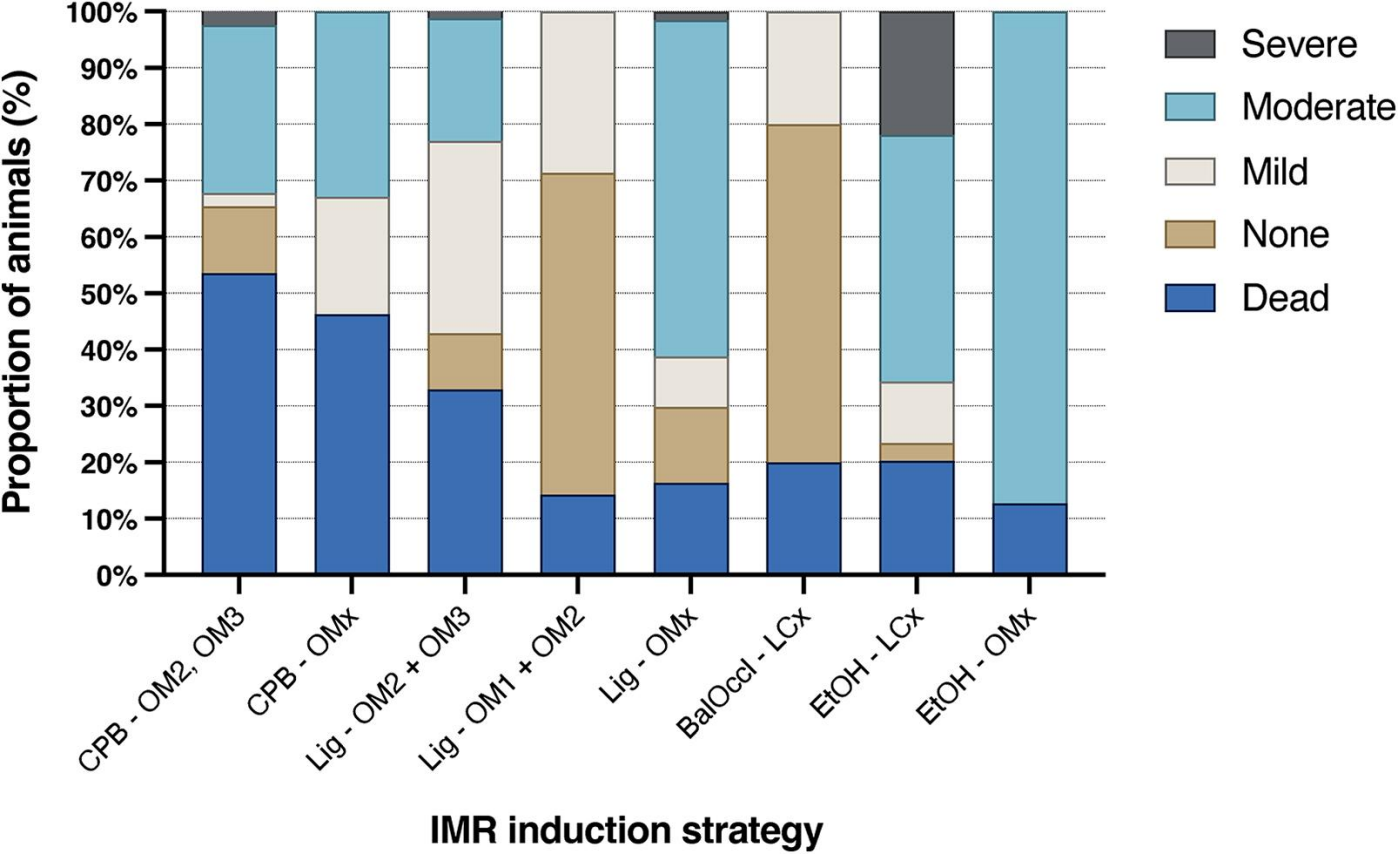
Stratification of ischemic mitral regurgitation severity by infarction strategy. Each IMR severity category represents the percentage of animals developing that grade relative to the total number of animals in the respective group. The “dead” category refers to the percentage of animals in each infarction strategy that died before the final echocardiographic assessment. BalOccl, balloon occlusion; CPB, cardiopulmonary bypass; EtOH, ethanol; LCx, left circumflex artery; Lig, ligation; OM1, OM2, OM3, obtuse marginal arteries 1, 2, and 3; OMx, select obtuse marginal arteries.

Figure 5 provides a graphical overview of IMR severity and associated mortality across infarction strategies. See Supplementary Table S4 for subgroup-specific data. The CPB-OM2, OM3, CPB-OMx, and Lig-OM2, OM3 strategies demonstrated the highest mortality rates, at 53.6%, 46.3%, and 32.9%, respectively. In contrast, the remaining strategy showed lower mortality rates, ranging from 12.7% to 20.3%, with the EtOH-OMx strategy exhibiting the lowest overall mortality rate. All infarction strategies, except Lig-OM1, OM2 and Balloon Occlusion-LCx, produced IMR of at least moderate severity. The Lig-OMx strategy resulted in moderate to severe IMR in 61.2% of animals. Among all methods, the EtOH-based strategy yielded the highest proportions of moderate-to-severe IMR: 65.7% for EtOH-LCx and 87.3% for EtOH-OMx. Among all evaluated methods, the EtOH-OMx strategy demonstrated the most favourable balance between low mortality and high reproducibility for moderate to severe IMR, and appears to be the most promising infarction strategy based on the currently available data.

#### Study heterogeneity

Given the small number of subgroups contributing to most infarction strategies, formal heterogeneity statistics were not applied, as they would not yield reliable or interpretable results. The qualitative subgroup heterogeneity analysis demonstrated considerable variability in IMR outcomes for several infarction strategies, including Lig-OM1, OM2, Lig-OM2, OM3, Balloon Occlusion-LCx, and EtOH-LCx. Within these groups, both the severity of IMR and the distribution of positive vs. adverse outcomes varied substantially between studies. In contrast, the subgroups using Lig-OMx, CPB-OMx, CPB-OM2, OM3, and EtOH-OMx displayed less intra-strategy heterogeneity across the same outcome measures. Given the reliance on visual inspection rather than formal heterogeneity statistics, these observations should be regarded as exploratory and hypothesis-generating rather than definitive. For a visual summary of these findings, see Supplementary Figure S1.

## Discussion

This systematic review is the first to comprehensively compare infarction strategies for inducing IMR in large-animal models. It highlights substantial variation in methodological approaches, inconsistencies in reporting quality, and notable heterogeneity in IMR outcomes across infarction strategies. Among these, the EtOH-OMx strategy demonstrated the highest and most consistent success rate in inducing moderate IMR. However, this finding should be interpreted cautiously, given the limited number of contributing subgroups and the overlap of confidence intervals with several comparator strategies. Given the number of pairwise comparisons performed, the risk of inflated type I error must be acknowledged, and the pooled proportions and confidence intervals should be prioritised over *p*-values when interpreting relative performance.

Furthermore, the robustness of this finding warrants cautious interpretation. The upper bounds of the 95% confidence intervals for Lig-OM2/OM3, Lig-OMx, and EtOH-LCx closely approximate the lower bound of EtOH-OMx, and substantial heterogeneity exists within these comparator groups. Additionally, the EtOH-OMx strategy could not be evaluated independently in sheep because only a single subgroup was available. As such, no definitive conclusion can be drawn regarding the comparability of the ovine and porcine models for this method, although the ovine study by Hamza et al. reported encouraging results (33).

There are significant procedural differences between infarction strategies—particularly among ligation (with or without CPB), balloon occlusion, and ethanol injection. First, ligation and balloon occlusion produce myocardial infarction strictly at the site of mechanical obstruction. In contrast, balloon occlusion followed by ethanol administration not only induces a thrombus but also introduces ethanol, a water-soluble, diffusible substance. This allows ethanol to extend tissue injury beyond the immediate occlusion site and potentially into downstream regions, thereby amplifying the ischemic effect. Second, percutaneous strategies naturally impose less surgical trauma, which may reduce non-cardiac mortality compared with open-chest ligation techniques. Finally, the percutaneous approaches depend on real-time identification of the arterial supply to the posteromedial papillary muscle, enabling more accurate targeting. In contrast, ligation techniques rely on predefined anatomical landmarks rather than functional imaging. Given that collateral blood supply is often overlooked in these models, the imaging-guided percutaneous strategies may mitigate this uncertainty more effectively than ligation approaches.

An additional consideration is whether the superior performance of the EtOH-OMx strategy arises primarily from the ethanol-based method, the anatomical choice of the OMx territory, or from their interaction. Ethanol injection differs fundamentally from ligation and balloon occlusion by inducing tissue injury beyond the immediate occlusion site through diffusion into adjacent and downstream myocardial regions, potentially intensifying papillary muscle dysfunction and diminishing collateral perfusion. At the same time, the OMx branches often provide dominant supply to the posteromedial papillary muscle, and targeting multiple marginal branches may create a more extensive and functionally relevant ischemic territory for IMR induction. Notably, ethanol injection into the LCx (EtOH-LCx) did not demonstrate superior performance compared with non-ethanol LCx-based methods, indicating that ethanol does not exert a uniform advantage across all coronary territories. Thus, the high reproducibility observed for EtOH-OMx likely reflects a combination of method-specific and territory-specific effects, which cannot be isolated with the currently available data. These observations and potential confounding from species differences limit the strength of any comparative conclusions.

In addition, the EtOH-OMx strategy may better replicate clinically relevant patterns of ventricular remodelling seen in human IMR. Unlike broad ligation-based approaches, which can create large, anatomically variable infarcts, the EtOH-OMx method produces a more localised, controlled injury to the vascular territories supplying the posteromedial papillary muscle. This targeted ischaemia reflects the predominant mechanism of human IMR, in which papillary muscle displacement and altered subvalvular geometry are central determinants of leaflet tethering. Moreover, the percutaneous nature of the EtOH-OMx model avoids the non-physiological surgical trauma inherent in open thoracotomy and cardiopulmonary bypass, thereby reducing confounding effects on ventricular remodelling. Collectively, these features may explain why the EtOH-OMx strategy yielded more consistent, higher-grade IMR across studies than other infarction methods. 

Several biological and methodological factors are likely to contribute to the heterogeneity observed across infarction strategies. Species differences between ovine and porcine models may influence susceptibility to ventricular fibrillation, ventricular remodelling patterns, and mitral apparatus geometry, thereby affecting both IMR incidence and mortality. Variation in infarct location and extent (for example, selective ligation of OM2/OM3 vs. broader OMx or LCx territory) is expected to produce different degrees of papillary muscle displacement and leaflet tethering. Differences in follow-up duration (4-16 weeks in the included studies) may also alter the balance between acute infarct-related dysfunction and chronic ventricular remodelling. Furthermore, heterogeneity in anaesthetic protocols, peri-procedural anti-arrhythmic regimens, and imaging strategies (including the use of two- vs. three-dimensional echocardiography and differing thresholds for RJ/LAA% or RF%) is likely to influence both the detection and grading of IMR. Owing to the limited number of subgroups in most infarction strategies, these potential contributors could be addressed only qualitatively, not through formal meta-regression.

No established risk-of-bias tools were applied due to the substantial heterogeneity in study aims, designs, and outcome measures among the included articles. Instead, we developed a bespoke reporting-quality questionnaire to evaluate the transparency and completeness of reporting across studies, rather than to assess internal validity. This approach enabled us to identify studies eligible for inclusion in the meta-analysis based on transparent reporting of mortality and IMR outcomes. Elements from the SYRCLE tool were incorporated where relevant. Still, additional items were required to capture methodological features specific to large-animal surgical models that are not addressed by existing tools.

Twelve of the 44 included studies (27.3%) were excluded from both the quantitative and severity analyses due to insufficient transparency regarding the outcomes of the included animals. Specifically, these studies failed to report whether IMR was successfully induced in each subject. Notably, the majority of these non-reporting studies employed the Lig-OM2/OM3 infarction strategy. These exclusions may have biased the relative performance of specific infarction strategies, particularly Lig-OM2/OM3, toward studies with more complete reporting, thereby underestimating the true variability of these models.

To date, ovine models are the most widely used for *in vivo* IMR research. However, no evidence supports the superiority of either the ovine or the porcine species for modelling IMR. Both models exhibit cardiovascular physiology that closely resembles that of humans, particularly in terms of baseline heart rate, blood pressure, contraction-relaxation dynamics, and coronary anatomy (16, 67). A shared limitation of both species is their susceptibility to ventricular fibrillation, which can be mitigated by appropriate monitoring, anaesthetic protocols, and premedication with anti-arrhythmic agents (68, 69). A specific challenge in long-term porcine studies is the rapid weight gain observed in conventional breeds. To address this, the use of minipigs has been recommended, as they maintain heart-to-body weight ratios comparable to those of humans and demonstrate greater resilience to surgical trauma and arrhythmias. Ultimately, the choice of animal model is often guided by institutional tradition and logistical feasibility, rather than clear biological advantages, given the current lack of comparative evidence favouring one species over the other in the context of IMR.

The assessment of reporting quality revealed several shortcomings across the included preclinical studies, particularly concerning animal sex, randomisation, and blinding of outcome assessors. Reporting of animal sex was frequently omitted, despite being straightforward to document. This is potentially relevant, as one human study using the MitraClip device identified female sex as the strongest predictor of left ventricular reverse remodelling following successful percutaneous mitral valve repair (70). Although this finding has not yet been validated in other randomised controlled trials, it underscores the importance of routinely reporting sex in preclinical research.

Randomisation was reported in only 44% of applicable studies (25, 26, 28, 32, 42–44, 50, 51, 58). Its consistent implementation and transparent reporting are essential for minimising bias and enhancing the translational value of preclinical findings. Blinding of echocardiographers to the intervention status of animals was also poorly reported, despite its critical role in evaluating qualitative outcomes, such as IMR severity. A total of 75% of studies (33/44) assessed IMR severity using regurgitation jet area as a percentage of the left atrial area (RJ/LAA%), or a related modification, whereas 25% (11/44) used regurgitation fraction (RF%). Among these, RJ/LAA% is inherently more subjective and prone to inter-observer variability (71). In the absence of blinding, there is an increased risk of differential misclassification, whereby echocardiographers may be more likely to assign higher IMR grades to intervention animals than to controls.

Furthermore, it should be emphasised that the questionnaire used in this review constitutes a reporting-quality assessment rather than a formal SYRCLE-based risk-of-bias analysis, as it combines SYRCLE items with additional reporting criteria and does not generate domain-specific risk-of-bias judgements. Finally, given the subjective nature of RJ/LAA% and its greater inter-observer variability, future studies should prioritise the use of RF% whenever feasible, as this measure is less dependent on qualitative interpretation. When RJ/LAA% is used, the implementation of blinded assessment, or preferably centralised core-lab evaluation, would help mitigate bias and improve the reliability of severity grading. Incorporating these practices would strengthen methodological rigour and enhance comparability across studies.

The findings of this study may facilitate a reduction in animal use by identifying infarction strategies with the highest success rates for inducing IMR, thereby improving experimental efficiency and supporting adherence to the 3Rs principle (72). In addition, they may assist researchers in selecting infarction methods that entail less surgical trauma, thereby contributing to more ethically and scientifically robust study designs.

### Limitations

A graphical representation of IMR severity within each infarction strategy was employed to assess heterogeneity. Conventional statistical measures (*I*^2^ or *χ*^2^) were not applied because the majority of infarction strategies were supported by only one or two subgroups, rendering these statistics mathematically unreliable and potentially misleading. In line with recommendations for meta-analyses with minimal replications, heterogeneity was therefore assessed qualitatively. This graphical approach is inherently qualitative, relies on subjective visual interpretation, and is thus susceptible to bias. Moreover, the absence of formal quantitative heterogeneity estimates means that the magnitude of between-study variability cannot be quantified, which represents a limitation of the present meta-analysis. Despite these limitations, the heterogeneity analysis remains a strength of the study, as it exposes internal inconsistencies within individual infarction strategies. However, it does not elucidate the underlying sources of heterogeneity.

The requirement for at least two subgroups per infarction strategy means the review is primarily informative for the most commonly used strategies. Niche or emerging methods, represented by only one eligible subgroup, could not be incorporated into the quantitative synthesis. Consequently, their actual performance characteristics remain uncertain and may differ from the aggregated results presented here.

No sensitivity analyses were conducted for echocardiographic modality or follow-up duration, owing to the limited number of eligible subgroups per infarction strategy. Such analyses would have provided valuable insights into the impact of these variables on both the incidence and severity of IMR and may have helped to identify additional contributors to the observed heterogeneity. The literature search was restricted to PubMed and Embase, and the review protocol was not prospectively registered, which may have reduced transparency and completeness in the identification of studies.

Finally, the reporting-quality assessment used in this review focuses on completeness of reporting rather than the definite presence or absence of specific methodological safeguards. Items scored as “no” frequently indicate that a given aspect was not reported, rather than that it was not performed. As a consequence, the summary of reporting quality may overestimate the true prevalence of methodological shortcomings across the included studies. Additionally, it should be noted that the tool applied in this review evaluates reporting quality rather than internal validity, and therefore does not constitute a formal risk-of-bias assessment.

To date, there are no established guidelines for evaluating IMR severity in animal models. However, the ovine and porcine mitral valve apparatus exhibits anatomical and physiological characteristics that closely resemble those of the human heart (73, 74). Consequently, IMR severity in this review was assessed according to the human echocardiographic criteria proposed by Zoghbi et al. (23). Because these criteria were used only as conceptual references, mapping heterogeneous study-specific definitions onto a unified four-category scale required assumptions. This process may introduce misclassifications, particularly where qualitative or non-standard thresholds were reported. As no validated animal-specific severity framework exists, these limitations should be considered when interpreting the pooled severity findings. Finally, the search was restricted to English-language publications, which introduces the risk of language bias. Relevant studies published in other languages may therefore have been missed, potentially limiting the completeness of the evidence base.

Substantial variation in animal age and weight across the included studies posed a challenge to standardisation. As a result, absolute IMR measurements—such as vena contracta width, effective regurgitant orifice area, and regurgitant volume—were considered inadequate, as they rely on a relatively uniform heart size, which cannot be assumed in animal models with diverse physiological profiles.

## Conclusion

This systematic review provides a comprehensive synthesis of all available studies that employ infarction strategies to induce IMR in large-animal models, with severity assessed using relative echocardiographic measures. The findings highlight substantial variability in experimental approaches and underscore persistent methodological shortcomings, particularly concerning the reporting of animal sex, randomisation, blinding, and control group use, as well as incomplete documentation of mortality and IMR outcomes in a subset of studies. Among the evaluated methods, the EtOH-OMx strategy was associated with a higher incidence of IMR and a high proportion of moderate-to-severe regurgitation, even when accounting for species differences. Based on these results, the EtOH-OMx strategy appears to be a promising and reproducible approach for inducing IMR in preclinical large animal models. However, these conclusions should be interpreted with caution, given the limited number of subgroups, overlapping confidence intervals, and potential species- and procedure-related differences. Additional constraints include the qualitative assessment of heterogeneity, the inability to synthesise rarely used infarction strategies quantitatively, and the restriction to English-language publications, which introduces a risk of language bias. Further standardisation of methodologies, more consistent use of quantitative and preferably less observer-dependent IMR grading (for example, RF% or blinded core-lab assessment when using RJ/LAA%), and improved reporting practices are essential to strengthen the translational value of future preclinical research in this field.

## Data Availability

The original contributions presented in the study are included in the article/Supplementary Material, further inquiries can be directed to the corresponding author.

## Supplementary material

Supplementary Table S1 Search strategy for PubMed and Embase. Both databases were searched on Nov 24th, 2022. The number of articles identified by each search string is given

Supplementary Table S2 Self-made questionnaire for quality assessment. All questions proposed are seen. Each question is marked to indicate whether it comes from SYRCLE, their signalling questions or is “Additional”.

Supplementary Table S3 Tabulated representation of results in Figure 4. MR development stratified to each infarction strategy expressed as the proportion of animals developing MR with the corresponding Standard Error (SE) and p-values compared to reference (Lig-OM2, OM3). The intra-infraction strategy range for each strategy is shown: Cardiopulmonary bypass. LCx: Circumflex artery. OM1, OM2, OM3; Obtuse marginal artery 1, 2 or 3. OMx: Select obtuse marginal arteries. Lig; Ligation. BalOccl; Balloon Occlusion. EtOH; ethanol

Supplementary Table S4 Subgroup and study-specific IMR severity. Data for each included study and subgroup in the meta-analysis and IMR-severity analysis with respect to: Infarction strategy, number of animals included, number of animals developing IMR and the associated proportion. Severity of IMR was divided into dead, none, mild, moderate or severe per subgroup, shown as the number of animals. CPB: Cardiopulmonary bypass. LCx: Circumflex artery. OM1, OM2, OM3; Obtuse marginal artery 1, 2 or 3. OMx: Select obtuse marginal arteries. Lig; Ligation. BalOccl; Balloon Occlusion. EtOH; ethanol

Supplementary Figure S1 Cumulative bar chart stratifying IMR severity in each infarction strategy. Intra-subgroup heterogeneity tables tabulated per infarction strategy. Animal IMR outcomes are categorised as “Dead”, “None”, “Mild”, “Moderate” or “Severe” and assigned a % of total animals developing the category. A) CPB-OM2, OM3. B) CPB-OMx. C) Lig-OM1, OM2. D) Lig-OM2, OM3. E) Lig-OMx. F) BalOccl-LCx. G) EtOH-LCx. H) EtOH-OMx. CPB: Cardiopulmonary bypass. LCx: Circumflex artery. OM1, OM2, OM3; Obtuse marginal artery 1, 2 or 3. OMx; Select obtuse marginal arteries. Lig; Ligation. BalOccl; Balloon Occlusion. EtOH; ethanol.
