# Supplementary material for: Large animal models of ischemic mitral regurgitation—systematic review and meta-analysis
Source: Front Med Technol. 2026 Jan 15;7:1687873. doi: 10.3389/fmedt.2025.1687873 (PMC12880048; doi:10.3389/fmedt.2025.1687873)
Supplement: Supplementary Table S1 — Search strategy for PubMed and Embase. Both databases were searched on Nov 24th, 2022. The number of articles identified by each search string is given [file Table1.docx]

***Supplementary Table 1: Search String***

| PubMed string | Search input | No. of articles |
| --- | --- | --- |
| String #1 | "Mitral valve insufficiency"[MeSH Terms] OR "Mitral Valve insufficien*" [tiab] OR "ischemic mitral valve regurgitation" [tiab] OR "ischemic mitral regurgitation" [tiab] OR "IMR" [tiab] OR "mitral valve prolapse" [tiab] OR "mitral valve regurgitation" [tiab] OR "mitral valve incompetence" [tiab] OR "mitral prolapse" [tiab] OR "myxomatous mitral valve" [tiab] OR "myxomatous mitral valve disease" [tiab] | 34,044 |
| String #2 | "Swine" [tiab] OR "porcine" [tiab] OR "pig" [tiab] OR "pigs" [tiab] OR "ovine" [tiab] OR "sheep" [tiab] OR "cattle" [tiab] OR “bovine” [tiab] OR "horses" [tiab] OR "Sus Scrofa" [Mesh] OR "Ruminants"[Mesh] OR ”goat”[tiab] | 959,872 |
| String #3 | "Models, Animal" [Mesh] OR "Disease models, Animal" [Mesh] OR "Proof of concept study" [Mesh] | 637,654 |
| Final Search PubMed | String #1 AND #2 AND #3 | 219 |

| Embase string | Search input | No. of articles |
| --- | --- | --- |
| String #1 | ’mitral valve regurgitation’/exp OR ’mitral valve regurgita*’:ti,ab,kw OR ’mitral valve insuffici*’:ti,ab,kw OR ’ischemic mitral valve regurgitation’:ti,ab,kw OR ’ischemic mitral regurgitation’:ti,ab,kw OR ’imr’:ti,ab,kw OR ’mitral valve prolapse’:ti,ab,kw OR ’mitral valve incompetence’:ti,ab,kw OR ’myxomatous mitral valve disease’:ti,ab,kw OR ’myxomatous mitral valve’:ti,ab,kw | 64,103 |
| String #2 | ’pig’:ti,ab,kw OR ’pigs’:ti,ab,kw OR ’sheep’:ti,ab,kw OR ’cow’:ti,ab,kw OR ’bovine’:ti,ab,kw OR ’horse’:ti,ab,kw OR ’sus scrofa’/exp OR ’goat’:ti,ab,kw OR 'ruminant'/exp | 1,026,125 |
| String #3 | ’Animal model’/exp OR ’disease models’/exp OR 'proof of concept study'/exp | 1,688,789 |
| Final Search Embase | String #1 AND #2 AND #3 | 345 |
