# Supplementary material for: Large animal models of ischemic mitral regurgitation—systematic review and meta-analysis
Source: Front Med Technol. 2026 Jan 15;7:1687873. doi: 10.3389/fmedt.2025.1687873 (PMC12880048; doi:10.3389/fmedt.2025.1687873)
Supplement: Supplementary Table S2 — Self-made questionnaire for quality assessment. All questions proposed are seen. Each question is marked to indicate whether it comes from SYRCLE, their signalling questions or is “Additional”. [file Table2.docx]

***Supplementary Table 2: Reporting Assessment Questionnaire***

|  | **Assessment of reporting quality questions** | **SYRCLE** | **SYRCLE (signalling questions)** | **Additional** |
| --- | --- | --- | --- | --- |
| Q1 | Was the animal species described? |  |  | X |
| Q2 | Was the strain of species described? |  |  | X |
| Q3 | Was age or weight of animals described? |  | X |  |
| Q4 | Number of animals described? |  |  | X |
| Q5 | Was the sex of animals declared? |  | X |  |
| Q6 | Was a control group described? |  | X | X |
|  | **Q6* - In case yes:** |  |  |  |
| Q6* | Was the number of animals per group described? |  |  | X |
| Q6* | Was randomization of animals into groups present and described? |  | X |  |
| Q6* | Were there comparable baseline values for groups? |  | X |  |
| Q6* | Was the final echocardiographic assessment done blinded? | X |  |  |
|  | **Q6* - In case no:** |  |  |  |
| Q6* | No, the study is an orginal animal model |  |  | X |
| Q6* | No, the study is a feasability/valvular geometric study |  |  | X |
|  |  |  |  |  |
| Q7 | Was the surgical MR-induction procedure clearly described? |  |  | X |
| Q8 | Was the timecourse of follow-up clearly described? |  |  | X |
| Q9 | Was the echocardiographic modality described? |  |  | X |
| Q10 | Were adverse events described? |  |  | X |
|  | **Q10** - In case yes:** |  |  |  |
| Q10** | Were the allocation of adverse events clearly described in a group(s)? |  | X |  |
| Q10** | Was the time point of adverse events clearly described? |  |  | X |
|  | **Q10** - In case no:** |  |  |  |
| Q10** | No, the author fails to "comment on adverse events" |  |  | X |
| Q10** | No, suspicion arise to author not including diseased animals in article |  |  | X |
