# Supplementary material for: Large animal models of ischemic mitral regurgitation—systematic review and meta-analysis
Source: Front Med Technol. 2026 Jan 15;7:1687873. doi: 10.3389/fmedt.2025.1687873 (PMC12880048; doi:10.3389/fmedt.2025.1687873)
Supplement: Supplementary Table S3 — Tabulated representation of results in Figure 4. MR development stratified to each infarction strategy expressed as the proportion of animals developing MR with the corresponding Standard Error (SE) and p-values compared to reference (Lig-OM2, OM3). The intra-infraction strategy range for each strategy is shown: Cardiopulmonary bypass. LCx: Circumflex artery. OM1, OM2, OM3; Obtuse marginal artery 1, 2 or 3. OMx: Select obtuse marginal arteries. Lig; Ligation. BalOccl; Balloon Occlusion. EtOH; ethanol [file Table3.docx]

Supplementary Table 3: Tabulated representation of results in Figure 4.

| **Infarction strategy** | Proportion (%) | SE (%) | p-values | Range in IMR development  Study reported  (Lowest - Highest) |
| --- | --- | --- | --- | --- |
| CPB-OM2,OM3 | 31.05 | 4.93 | <0,001 | 27,8 % - 38,5% |
| CPB-OMx | 32.81 | 5.68 | <0,001 | 27,9% - 41,6% |
| Lig-OM2,OM3 | 55.88 | 3.81 | 1 (ref) | 25,8% - 100% |
| Lig-OM1,OM2 | 14.59 | 9.32 | <0,001 | 0% - 16,7% |
| Lig-OMx | 61.19 | 5.86 | 0,447 | 46,2% - 71,4% |
| BalOccl-LCx | 20.01 | 8.19 | <0,001 | 0% - 50% |
| EtOH-LCx | 62.48 | 5.20 | 0,318 | 40% - 89,6% |
| EtOH-OMx | 87.26 | 4.28 | <0,001 | 64,3% - 100% |
| EtOH-OMx (pigs) | 85.42 | 4.68 | <0,001 | 64,3% - 96% |
