# Supplementary material for: Large animal models of ischemic mitral regurgitation—systematic review and meta-analysis
Source: Front Med Technol. 2026 Jan 15;7:1687873. doi: 10.3389/fmedt.2025.1687873 (PMC12880048; doi:10.3389/fmedt.2025.1687873)
Supplement: Supplementary Table S4 — Subgroup and study-specific IMR severity. Data for each included study and subgroup in the meta-analysis and IMR-severity analysis with respect to: Infarction strategy, number of animals included, number of animals developing IMR and the associated proportion. Severity of IMR was divided into dead, none, mild, moderate or severe per subgroup, shown as the number of animals. CPB: Cardiopulmonary bypass. LCx: Circumflex artery. OM1, OM2, OM3; Obtuse marginal artery 1, 2 or 3. OMx: Select obtuse marginal arteries. Lig; Ligation. BalOccl; Balloon Occlusion. EtOH; ethanol [file Table4.docx]

***Supplementary Table 4: Subgroup and study-specific IMR severity***

| **Author** | # animals | #develops IMR | Proportion of IMR  Self-reported | Dead | None | Mild | Moderate | Severe |
| --- | --- | --- | --- | --- | --- | --- | --- | --- |
| **CPB - OM2, OM3** | |  |  |  |  |  |  |  |
| Tibayan 2005 | 26 | 10 | 38,46 % | 13 | 3 | 0 | 10 | 0 |
| Tibayan 2003 | 36 | 10 | 27,78% | 19 | 7 | 0 | 10 | 0 |
| Rausch 2013 | 25 | 7 | 28% | 13 | 0 | 2 | 5 | 2 |
| **CPB - OMx** | |  |  |  |  |  |  |  |
| Tibayan 2003 | 24 | 10 | 41,67% | 8 | 0 | 6 | 10 | 0 |
| Tibayan 2004 | 43 | 12 | 27,9% | 23 | 0 | 8 | 12 | 0 |
| **Lig - OM1, OM2** | |  |  |  |  |  |  |  |
| Quick 1997 | 2 | 0 | 0% | 0 | 0 | 2 | 0 | 0 |
| Llaneras 1993 | 12 | 2 | 16,67% | 2 | 8 | 2 | 0 | 0 |
| **Lig-OM2, OM3** | |  |  |  |  |  |  |  |
| Castillero 2021 | 14 | 6 | 42,86% | 4 | 4 | 6 | 0 | 0 |
| Messas 2006 | 13 | 13 | 100% | 0 | 0 | 13 | 0 | 0 |
| Quick 1997 | 4 | 2 | 50% | 0 | 0 | 2 | 2 | 0 |
| Szymanski 2012 | 62 | 37 | 59,68% | 25 | 0 | 37 | 0 | 0 |
| Daimon 2005 | 31 | 8 | 25,81% | 12 | 11 | 0 | 8 | 0 |
| Messas 2010 | 30 | 18 | 60% | 12 | 0 | 0 | 18 | 0 |
| Llaneras 1993 | 16 | 11 | 68,75% | 3 | 2 | 0 | 9 | 2 |
| **Lig-OMx** | |  |  |  |  |  |  |  |
| Nguyen 2007 | 14 | 6 | 46,15% | 1 | 7 | 0 | 6 | 0 |
| Matsuzaki 2010 | 33 | 20 | 60,61% | 7 | 0 | 6 | 20 | 0 |
| Rodell 2020 | 8 | 5 | 62,5% | 1 | 2 | 0 | 4 | 1 |
| Robb 2011 | 14 | 10 | 71,43% | 4 | 0 | 0 | 10 | 0 |
| **BalOccl-LCx** | |  |  |  |  |  |  |  |
| Ishikawa 2014 | 6 | 3 | 50% | 1 | 2 | 3 | 0 | 0 |
| Ishikawa 2018 | 9 | 0 | 0% | 2 | 7 | 0 | 0 | 0 |
| **EtOH-LCx** | |  |  |  |  |  |  |  |
| Pasrija 2021 | 35 | 14 | 40% | 12 | 0 | 7 | 2 | 14 |
| Xu 2022 | 29 | 26 | 89,66% | 1 | 2 | 0 | 26 | 0 |
| **EtOH-OMx** | |  |  |  |  |  |  |  |
| Shi 2017 | 14 | 9 | 64,29% | 5 | 0 | 0 | 9 | 0 |
| Hamza 2019 | 8 | 7 | 87,5% | 1 | 0 | 0 | 7 | 0 |
| Hamza 2020 | 7 | 7 | 100% | 0 | 0 | 0 | 7 | 0 |
| Sarin 2016 | 26 | 25 | 96,15% | 1 | 0 | 0 | 25 | 0 |
