# Supplementary figures and images for: Large animal models of ischemic mitral regurgitation—systematic review and meta-analysis
Source: Front Med Technol. 2026 Jan 15;7:1687873. doi: 10.3389/fmedt.2025.1687873 (PMC12880048; doi:10.3389/fmedt.2025.1687873)

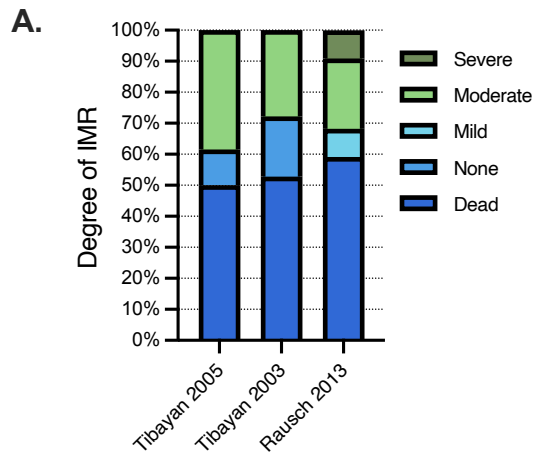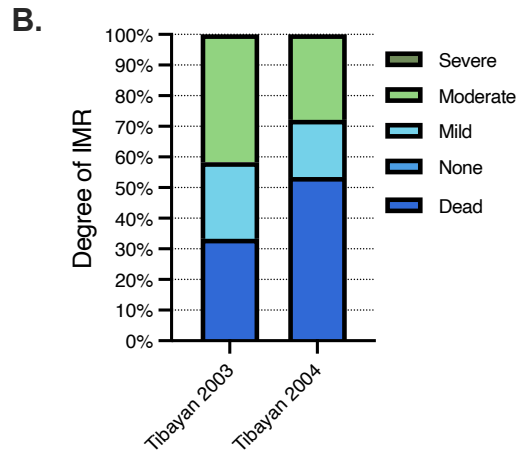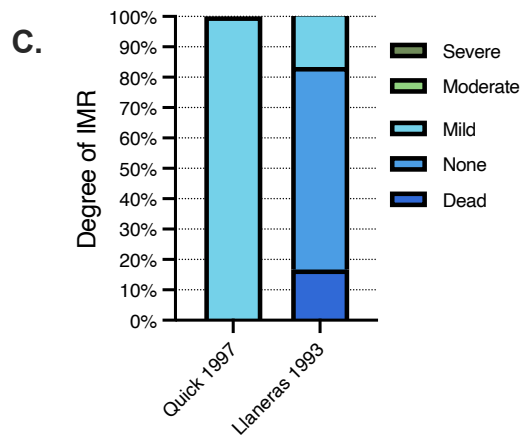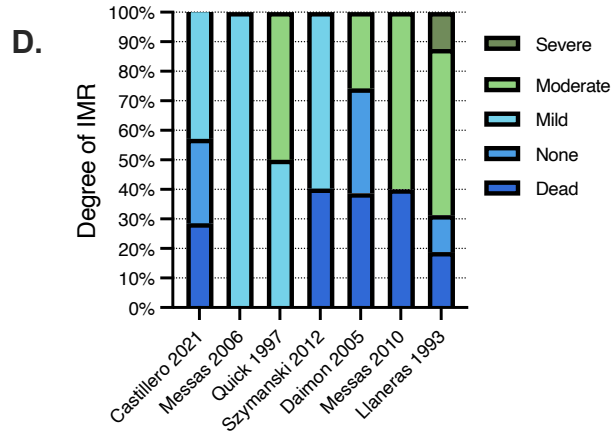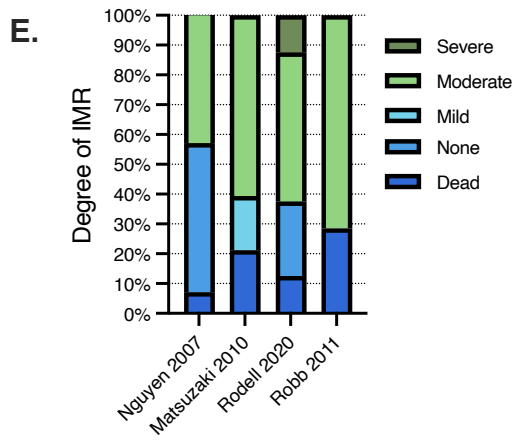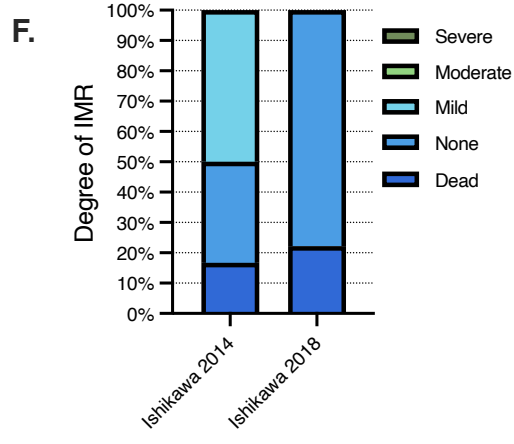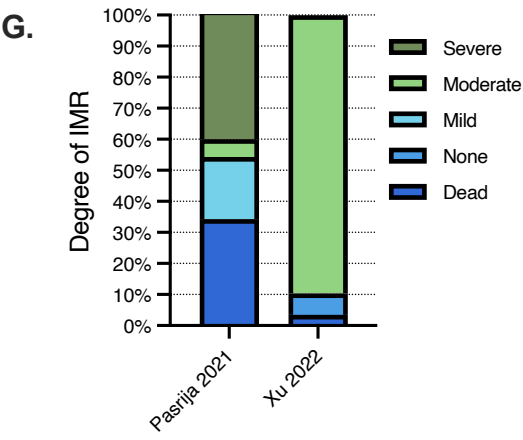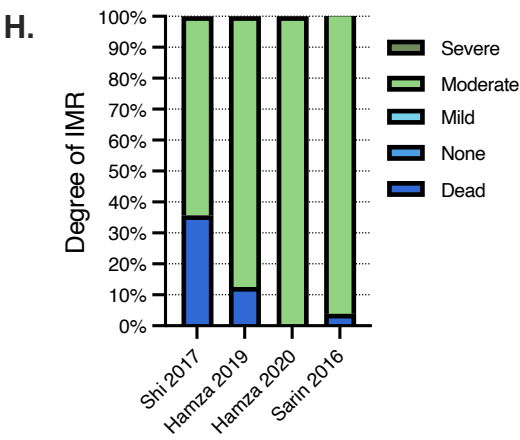

Supplement: Supplementary Figure S1 — Cumulative bar chart stratifying IMR severity in each infarction strategy. Intra-subgroup heterogeneity tables tabulated per infarction strategy. Animal IMR outcomes are categorised as “Dead”, “None”, “Mild”, “Moderate” or “Severe” and assigned a % of total animals developing the category. A) CPB-OM2, OM3. B) CPB-OMx. C) Lig-OM1, OM2. D) Lig-OM2, OM3. E) Lig-OMx. F) BalOccl-LCx. G) EtOH-LCx. H) EtOH-OMx. CPB: Cardiopulmonary bypass. LCx: Circumflex artery. OM1, OM2, OM3; Obtuse marginal artery 1, 2 or 3. OMx; Select obtuse marginal arteries. Lig; Ligation. BalOccl; Balloon Occlusion. EtOH; ethanol. [file Datasheet1.pdf]
